# Age-related changes in postural control in older women: transitional tasks in step initiation

**DOI:** 10.1186/s12877-020-01985-y

**Published:** 2021-01-06

**Authors:** Justyna Michalska, Anna Kamieniarz, Grzegorz Sobota, Magdalena Stania, Grzegorz Juras, Kajetan J. Słomka

**Affiliations:** Institute of Sport Sciences, Academy of Physical Education in Katowice, Katowice, Poland

**Keywords:** step initiation, posturography, crossing obstacle, stair negotiation

## Abstract

**Background:**

Aging, being a natural process, involves many functional and structural changes within the body. Identifying the age-related postural changes will provide insight into the role of aging on postural control during locomotion. The aim of this study was to identify age-related postural changes during a transitional task under different conditions.

**Methods:**

Sixty healthy females divided into three age groups: A (50-60 y/o), B (60-70 y/o), and C (70-80 y/o). The transitional task was measured by two force platforms. The procedure consisted of three phases: quiet standing, transfer onto a second platform, and quiet standing on the second platform. Four different conditions were applied: unperturbed transfer, obstacle crossing, step-up, and step-down. Double-support time, transit time, and stability time before and after the step task were analyzed.

**Results:**

The transit time was longer by 30% for subjects over 70 y/o. The double-support time was longer by 11% among adults 60-70 y/o, while in people over 70 y/o it was longer by almost 50% compared to the 50-60 y/o subjects. The stability time before the transitional task was longer by 17% among adults over 60 y/o compared to middle-age subjects. The stability times before and after the transitional task were longer for adults in the 50-60 y/o category.

**Conclusion:**

The proposed procedure is adequate for assessing age-related changes in postural control while undergoing a transitional task. An analysis of the double-support time and stability time before and after the step task enabled the detection of early signs of balance changes in middle-age adults. Independent of age, the transitional task parameters changed with the increasing difficulty of the tasks.

## Background

The maintenance of balance requires the interaction and coordination of several systems, such as sensory, musculoskeletal, and neuromuscular systems. Aging changes in postural control can be explained by deterioration in these systems. These changes involve reduced muscle strength [1], body deformities and inclined posture [2], impaired cognitive function, and declined motor responses [3]. In addition, deterioration in visual and vestibular systems decrease sense of orientation and awareness of the body, which increase the motor reaction time and affect the movement coordination [4]. Abovementioned issues impair the body balance in older people. In addition, the older adults present gait deterioration, such as changes in walking rhythm, shortening of the step length, and decreases in gait speed. These factors, combined with age-related lower activity levels, lead to postural instability and an increased risk of falls. These falls may occur during daily activities [5], which is a major problem as the consequences might induce a loss of mobility, decreased independence and quality of life, or even increased mortality among the older population. There is evidence that one of the main factors influencing falling is poor balance [6]. Changes in postural control, which lead to a balance deficit, appear prior to a fall incident. Therefore, an early and proper balance diagnostic is crucial in preventing serious injuries as a consequence of falls.

Balance can be assessed using either clinical balance tests such as the Tinetti test, Berg Balance Scale, Timed Up and Go, and Functional Reach test or more objective measures such as posturography. However, clinical tests only assess visible balance deficits, and they cannot always be used as a preventive diagnostic. According to Boulgarides et al. [7] the results of some subjective tests, combined with health and demographic factors, were insufficient to predict falls among the community-dwelling older adults. Therefore, developing a more sensitive testing procedure to measure postural changes, which are undetectable by standard clinical tests, is reasonable.

The older adults experience changes in the neuromuscular system with age, which is visible in the changes in gait pattern characteristics. The step length becomes narrower, the variability of the step length increases, gait speed and cadence slows, and double support time lasts longer [8, 9]. In addition, some authors claimed that falls occur predominantly during short distance movements [10]. For example, the older people have balance control problems during a transitional task, including step initiation, which is a common everyday activity. It is well-known that everyone must adjust to varying environmental constraints (i.e., ground unevenness and/or a curb) and/or stair negotiation. There is evidence, that stepping over an obstacle is tough [11]. However, the stair negotiation is the most difficult task due to old age, and it can be a very demanding for older adults which can even lead to falls [12]. As falls and the resulting injuries are one of the most serious health concerns that face the older adults, it is important that we understand how the changes in postural control may affect the locomotor tasks. Thus, we examined step initiation under four different transitional task conditions (unperturbed crossing, obstacle crossing, step-up and step-down).

The World Health Organization consider the onset of old age as occurring at 65 y/o. However, changes in postural control might appear earlier at 60 y/o [13, 14]. Thus, the following becomes an important question: “How quickly do these changes progress with age?” Several studies compared different age groups, and the authors presented that the most postural control problems occur between 60 – 70 y/o and became more pronounced after the age of 70 years [14, 15]. Nevertheless, some authors observed differences between the middle-aged groups (30–39, 40–49 and 50–59 years). Therefore, in our study the participants were divided into three decades, 50-60, 60-70, and 70-80 y/o, to determine the dynamic of these changes. Identifying the age-related postural changes will provide insight into the role of aging on postural control during locomotion.

Therefore, the aim of our study was to identify the postural changes that appear with age during a transitional task. We hypothesized that the step-up and step-down conditions are the most difficult of the transitional tasks, especially for people over 60 y/o, thus all analyzed posturographic parameters would increase with age.

## Methods

Sixty females voluntarily participated in the study. They were divided into three age groups: A (50-60 y/o), B (60-70 y/o), and C (70-80 y/o) (Table 1). The inclusion criteria included a minimum age of 50 and maximum age of 80. The exclusion criteria were as follows: severe neurological, cognitive impairments, or lower limb injuries. One of the subjects in group C failed to complete the entire research procedure. All women were independent and physically active. Group A constituted professionally active persons. Subjects from other groups were recruited from the local University of Third Age. The subjects voluntarily participated in the study, and they provided written informed consent. The research was approved by the Institutional Ethics Committee of the Medical University of Warsaw (number KB/28/2014).

**Table 1:** Characteristics of participants

	Group A	Group B	Group C	*p* value
Sample size	20	20	19	-
Age (y)	54.5 ± 3.5	66.5 ± 2.0	73.5 ± 2.5	< 0.05
Height (cm)	164.6 ± 6.8	160.6 ± 6.2	160.8 ± 6.0	> 0.05
Weight (kg)	68.0 ± 9.0	68.2 ± 10.7	69.8 ± 8.5	> 0.05

**Fig. 1:**
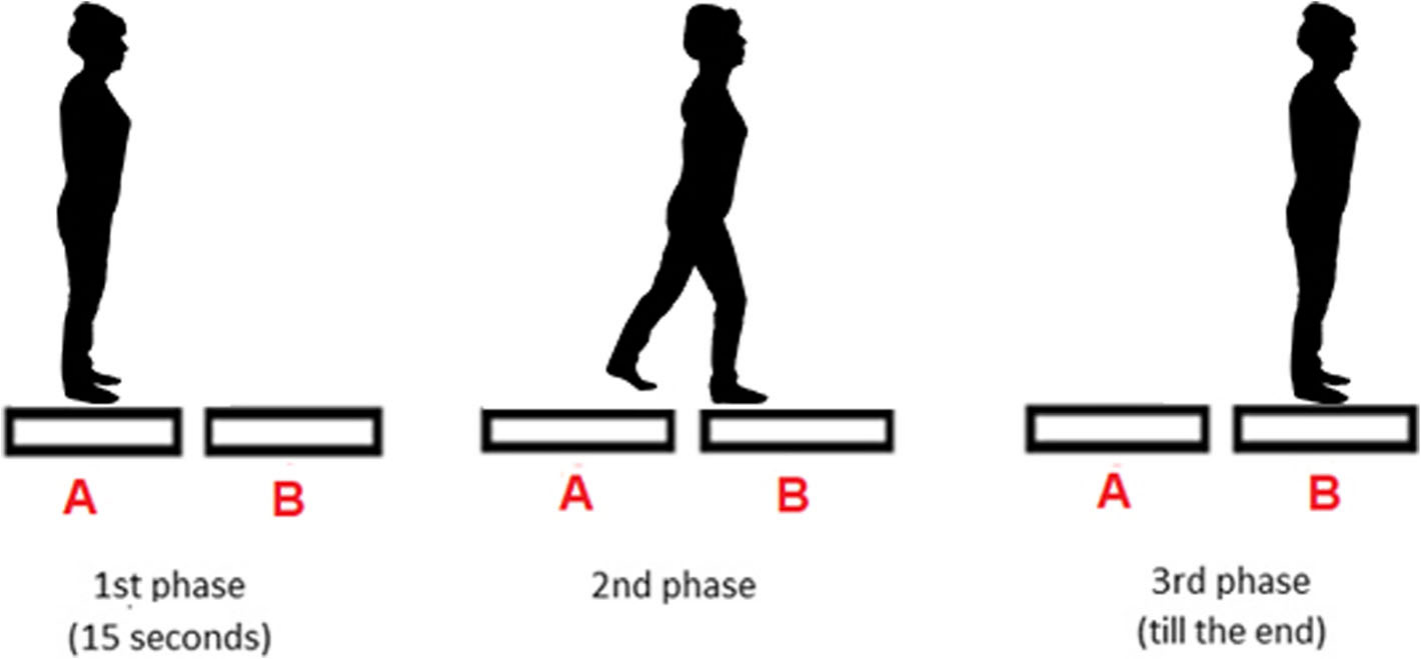
Experimental set-up – three phases [16]

The procedure of transitional task applied in this study have already been published in our previous studies [16, 17]. Measurements of the transitional task were performed by using two force platforms (AMTI, Accugait, Watertown, MA, USA) that registered the ground reaction forces and moments at a 100 Hz sampling frequency. The off-line raw data were processed using MATLAB software (Mathworks, Natic, MA). The data were low-pass filtered with a cut-off frequency of 7 Hz (low-pass Butterworth filter). The platforms (50 cm x 50 cm, height: 4.5 cm) were placed in a one line in front of each other, and the distance between the force plates was 4 cm (Fig. 1). This distance was dictated by the thickness of the obstacle [16, 17].

**Fig. 2:**
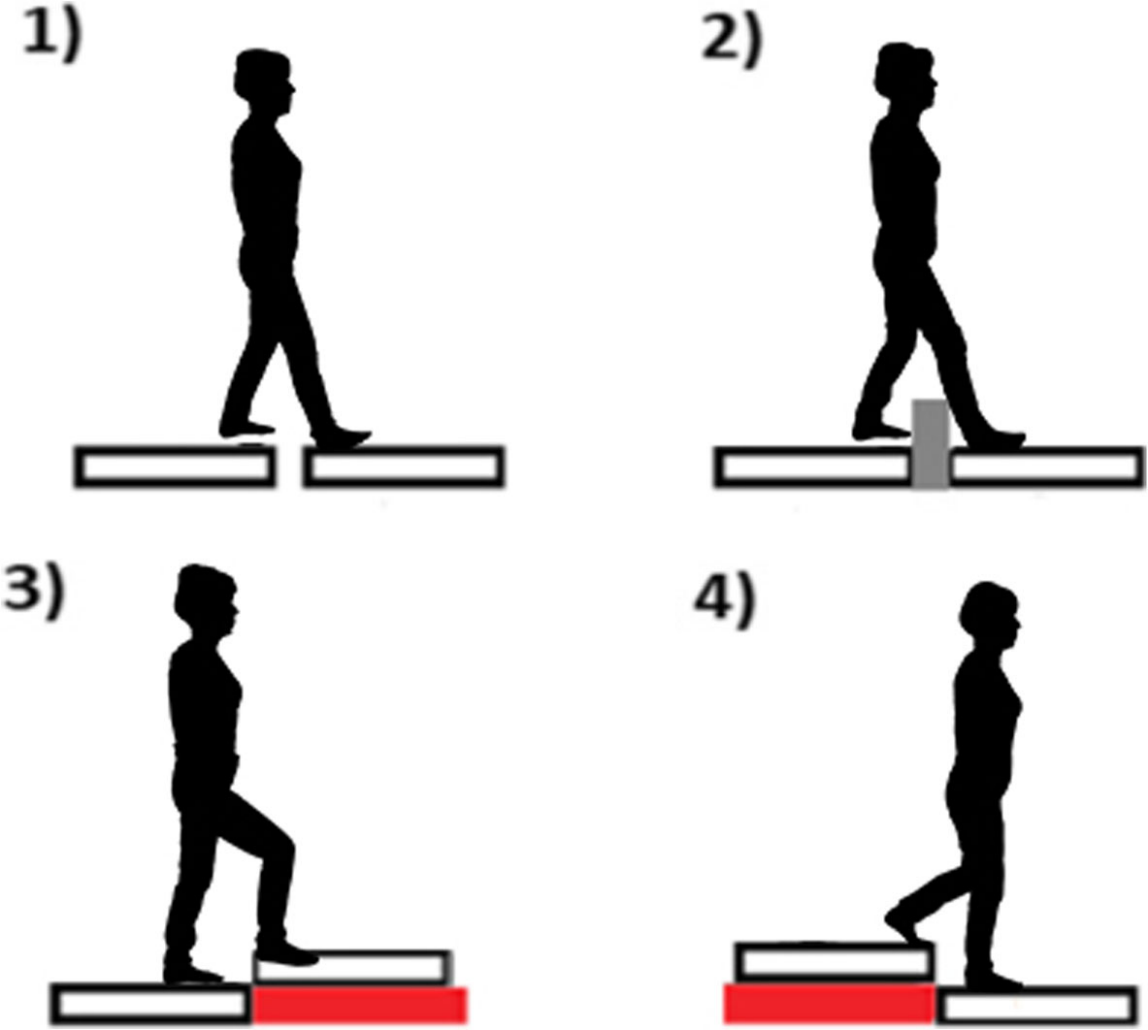
Four conditions of the transitional task [16]

Participants started all trials with a quiet standing with the feet positioned shoulder-width apart, arms alongside the body. The trial started with 15 seconds of quiet standing, followed by an acoustic signal for subjects to transfer from one to another platform, and after the transitional phase subjects performed quiet standing until the end of the trial (Fig. 1). The transitional task was executed under four different conditions: unperturbed crossing, crossing obstacle, and step-up and step-down (Fig. 2) [16, 17]. A 16cm/4cm (height /width) wooden block was used as an obstacle. In the step-up and step-down conditions, one platform was placed on a 17-cm base directly at the edge of the other platform. The placing of platforms were adopted based on the typical height of a curb and a stair step according to Polish building norms (6 – 16 cm and 15 – 17 cm, respectively) [16, 17].

The trials were repeated 3 times, lasted 35 s each, and the order of the trials was randomized. Before being examined, the subjects practiced the transitional task on the force platforms. The participants started all trials with their dominant leg and performed the step as quick as possible [16].

The calculation of the total momentary COP displacement (TMD_COP_) from both platforms allowed to divide the test into 3 phases. The first phase (quiet standing on the first platform), lasts from the beginning of the measurement to about 15 seconds. From the initial five seconds of this phase, the mean (Mf1_COP_) and standard deviation (SDf1_COP_) of TMD_COP_ were determined, which were used to determine the beginning of 2^nd^ phase (transit phase) (moment of time when: *TMD*_*COP*_ > *Mf*1_*COP*_ + 3 ∗ *SDf*1_*COP*_). The start of the 3^rd^ phase (quiet standing until the end of the measurement) was calculated based on the mean (Mf3_COP_) and the standard deviation (SDf3_COP_) of TMD_COP_ from the last 5 seconds of the measurement (moment of time when: *TMD*_*COP*_ < *Mf*3_*COP*_ + 3 ∗ *SDf*3_*COP*_). In addition, based on the vertical forces from both platforms (Fz_plat1_, Fz_plat2_), two characteristic times were determined: stability time 1 (S1) and stability time 2 (S2) (see Juras et al. [16]).

The following variables were analyzed [16, 17]:

1. 1^st^ Phase and 3^rd^ Phase:

- v-COP – the antero-posterior (AP) COP velocity (cm/s)
- std-COP – the antero-posterior (AP) standard deviation of COP (cm)

2. 2^nd^ Phase:

- TT (s) – transit time, the duration of the transit phase;
- S1 (s) – stability time 1 (preparatory stability time), time from the start of the 2^nd^ phase to the first foot contact with the second platform (*Fz*_*plat*2_ > 0);
- S2 (s) – stability time 2 (regained stability time), time from lifting the foot off the first platform (*Fz*_*plat*2_ = 0) to the start of the 3^rd^ phase;
- DST (s) – double-support time, when each foot is in contact with one of the platforms.

The Shapiro-Wilk test was used to check the data for a normal distribution. Variance homogeneity was checked with Levene’s test. The Kruskal-Wallis test was conducted to compare data between the three groups. Differences between the different transitional task conditions were determined by Friedman’s ANOVA. The level of significance was set at p *≤* 0.05. All calculations were carried out using STATISTICA v.13.1 (StatSoft, Inc., USA).

## Results

### Intergroup comparison between the three groups

There was no group effect on 1^st^ phase in unperturbed crossing, crossing the obstacle, and step-up and step-down conditions (p > 0.05).

There was a group effect on transit phase in unperturbed crossing, crossing the obstacle, and step-up conditions (Table 2). The greatest differences were observed between group A and C. Group C presented significantly higher values for TT, S1, S2, and DST under unperturbed crossing, crossing the obstacle, and step-up conditions compared to group A. Additionally, group B obtained significantly higher values of TT and S2 relative to group A while crossing the obstacle. Group B also showed significantly higher values of S1 and S2 during the step-up condition compared to group A. The least differences were observed between group B and C. Group B presented significantly lower value only in DST during unperturbed crossing, crossing obstacle compared to group C. There were no significant differences between groups during the step-down condition.

**Table 2.** Intergroup comparison between three groups A, B, and C

V	P	C	group A	group B	group C	H & p value	Intergroup comparison		
			Mdn (min-max)	Mdn (min-max)	Mdn (min-max)		A vs. B	B vs. C	A vs. C
v-COP [cm/s]	F I R S T	unperturbed crossing	0.82 (0.50-1.42)	0.94 (0.55-4.13)	0.85 (0.38-2.60)	H = 2.15 p = .3401	-	-	-
		obstacle crossing	0.76 (0.50-3.66)	1.08 (0.57-3.23)	1.00 (0.53-1.66)	H = 3.59 p = .1665	-	-	-
		step-up	0.88 (0.46- 1.70)	1.10 (0.51-3.38)	1.08 (0.59-2.33)	H = 4.67 p = .0967	-	-	-
		step-down	3.28 (0.93-1.87)	1.17 (0.63-5.84)	0.92 (0.48-1.65)	H = 2.75 p = .2529	-	-	-
std-COP [cm]		unperturbed crossing	0.37 (0.23-0.64)	0.42 (0.26-0.85)	0.36 (0.22-0.86)	H = 1.37 p = .5032	-	-	-
		obstacle crossing	0.37 (0.23-7.63)	0.46 (0.26-0.82)	0.42 (0.22-0.76)	H = 1.78 p = .4098	-	-	-
		step-up	0.40 (0.26- 0.60)	0.47 (0.25-0.87)	0.47 (0.23-0.92)	H = 2.37 p = .3057	-	-	-
		step-down	0.42 (0.26-0.87)	0.47 (0.26-1.10)	0.41 (0.20-1.16)	H = 2.96 p = .2273	-	-	-
TT [s]	S E C O N D	unperturbed crossing	2.70 (2.35-3.85)	3.32 (2.69-4.50)	3.57 (2.88-4.89)	H = 17.97 p = .0001	+	-	+
		obstacle crossing	3.01 (2.51-3.91)	3.45 (2.845-4.63)	3.48 (2.94-6.54)	H = 15.78 p = .0004	+	-	+
		step-up	2.93 (2.50- 4.50)	3.50 (2.64-4.71)	3.77 (3.07-5.83)	H = 13.56 p = .0011	-	-	+
		step-down	3.28 (2.53-4.44)	3.30 (2.65-5.10)	3.68 (3.05-5.06)	H = 5.77 p = .0559	-	-	-
S1 [s]		unperturbed crossing	1.11 (0.95-2.7)	1.29 (1.02-1.73)	1.37 (1.05-2.18)	H = 8.37 p = .0153	-	-	+
		obstacle crossing	1.30 (1.02-2.03)	1.36 (1.05-1.93)	1.47 (0.97-2.53)	H = 4.76 p = .0926	-	-	-
		step-up	1.12 (0.97-2.39)	1.27 (1.02-2.14)	1.35 (1.03-2.24)	H = 12.77 p = .0017	+	-	+
		step-down	1.32 (1.03-2.80)	1.37 (1.15-2.78)	1.50 (1.28-2.33)	H = 5.46 p = .0654	-	-	-
S2 [s]		unperturbed crossing	1.28 (0.82-2.51)	1.67 (1.19-2.24)	1.74 (1.30-2.74)	H = 12.87 p = .0016	-	-	+
		obstacle crossing	1.40 (1.03-2.13)	1.72 (1.44-2.36)	1.83 (1.40-3.60)	H = 16.56 p = .0003	+	-	+
		step-up	1.46 (1.17-2.45)	1.82 (1.19-2.88)	1.95 (0.99-3.09)	H = 11.82 p = .0027	+	-	+
		step-down	1.58 (1.31-2.12)	1.63 (1.09-2.94)	1.82 (1.41-3.40)	H = 5.85 p = .0536	-	-	-
DST [s]		unperturbed crossing	0.27 (0.15-0.44)	0.32 (0.23-0.47)	0.40 (0.26-0.71)	H = 18.19 p = .0001	-	+	+
		obstacle crossing	0.27 (0.17-0.40)	0.27 (0.22-0.54)	0.36 (0.28-0.61)	H = 22.14 p = .0000	-	+	+
		step-up	0.34 (0.19-0.91)	0.40 (0.26-0.79)	0.50 (0.25-0.79)	H = 8.85 p = .0120	-	-	+
		step-down	0.22 (0.13-0.66)	0.24 (0.18-0.41)	0.21 (0.07-0.34)	H = 4.47 p = .1068	-	-	-
v-COP [cm/s]	T H I R D	unperturbed crossing	0.61 (0.43-1.33)	0.84 (0.55-3.06)	0.76 (0.37-1.62)	H = 11.36 p = .0034	+	-	-
		obstacle crossing	0.59 (0.38-1.27)	0.88 (0.50-3.47)	0.81 (0.44-1.62)	H = 10.67 p = .0048	+	-	+
		step-up	0.73 (0.50-2.08)	0.89 (0.58-4.94)	0.92 (0.37-1.81)	H = 4.91 p = .0860	-	-	-
		step-down	0.64 (0.39-1.50)	0.91 (0.51-3.70)	0.90 (0.49-1.85)	H = 10.28 p = .0059	+	-	+
std-COP [cm]		unperturbed crossing	0.33 (0.19-0.50)	0.46 (0.23-0.74)	0.37 (0.20-0.76)	H = 6.10 p = .0473	+	-	-
		obstacle crossing	0.28 (0.18-0.56)	0.41 (0.20-0.68)	0.40 (0.24-0.76)	H = 10.27 p = .0059	+	-	+
		step-up	0.32 (0.16-0.54)	0.42 (0.16-1.05)	0.42 (0.22-1.33)	H = 6.42 p = .0403	-	-	-
		step-down	0.37 (0.21-0.63)	0.37 (0.23-0.92)	0.39 (0.18-1.16)	H = 0.37 p = .8298	-	-	-

*Abbreviations: V* variables, *P* phase, *C* condition, *TT* transit time, *S1* stability time 1, *S2* stability time 2, *DST* double support time, *group A* 50-60 years old, *group B* 60-70 years old, *group C* 70-80 years old, *‘+’* statistically significant differences between groups (*p* < 0.05); ‘-‘ – no statistically significant differences between groups (*p* > 0.05)

There was a group effect on 3^rd^ phase in unperturbed crossing, crossing the obstacle, and step-down conditions. The greatest differences were observed between group A and B. Group B presented significantly higher values for v-COP and std-COP under unperturbed crossing and crossing the obstacle conditions compared to group A. During step-down condition group B obtained significantly higher values of v-COP compared to group A. There were no significant differences between groups during the step-up condition.

### Intragroup comparisons during the 1^st^ phase

There was no significant impact of testing conditions on v-COP, and std-COP in all three groups in the first phase (p > 0.05).

### Intragroup comparisons during the 2^nd^ phase

**Fig. 3:**
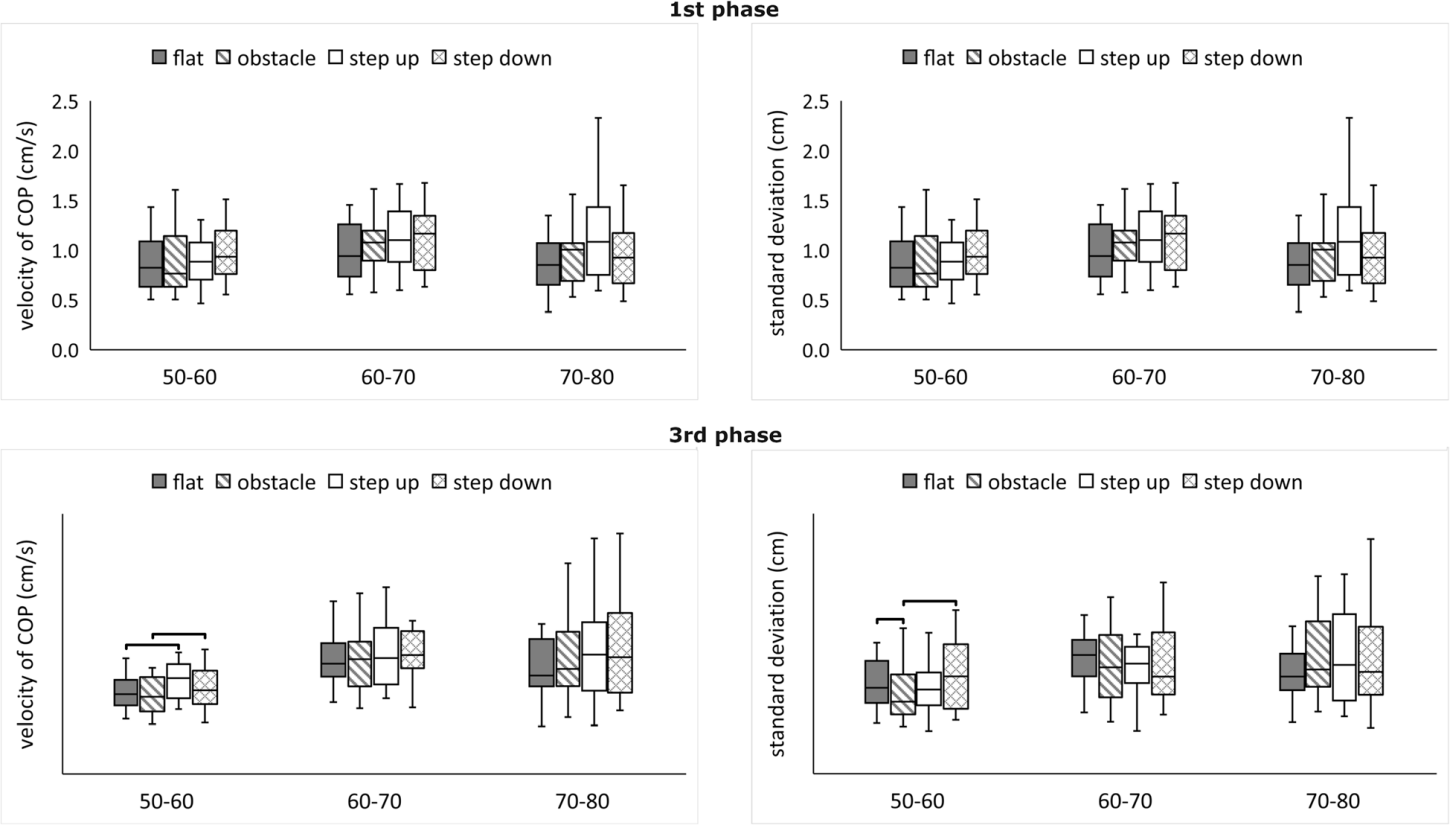
Median value of transit time, stability time 1, stability time 2, double-support time (minimum, maximum marked as error bars) in the four conditions (2^nd^ phase) among groups A, B, and C. The horizontal bars indicate statistically significant differences within the groups (Friedman ANOVA repeated measures with the post hoc test). Abbreviations: flat – unperturbed crossing; obstacle – perturbed crossing

There was a significant effect with regard to the testing conditions on TT (Chi2 = 16.74; *p* < 0.001), S1 (Chi2 = 22.53; *p* < 0.001), S2 (Chi2 = 11.70; *p* < 0.008), and DST (Chi2 = 34.74; *p*< 0.001) in group A. The subjects presented the longest TT, S1, and S2 during the step-down condition. At the same time, the results showed significantly higher values for DST during the step-up condition relative to other conditions (Fig. 3).

There was a significant effect with regard to the testing conditions on TT (Chi2 = 9.54; *p* < 0.05), S1 (Chi2 = 16.32; *p* < 0.001), S2 (Chi2 = 11.10; *p* < 0.05), and DST (Chi2 = 42.31; *p* < 0.001) in group B. Group B obtained the highest values of TT and S2 during the step-up condition. The same changes were observed when analyzing the DST variable, whereas the highest S1 values were achieved among subjects during the step-down condition (Fig. 3).

There was no significant effect with regard to the testing conditions on all analyzed variables except for DST (Chi2 = 41.04; *p* < 0.001) in group C. The subjects obtained the longest time for DST during the step-up condition and the shortest time for DST during the step-down condition (Fig. 3).

### Intragroup comparisons during the 3^nd^ phase

**Fig. 4:**
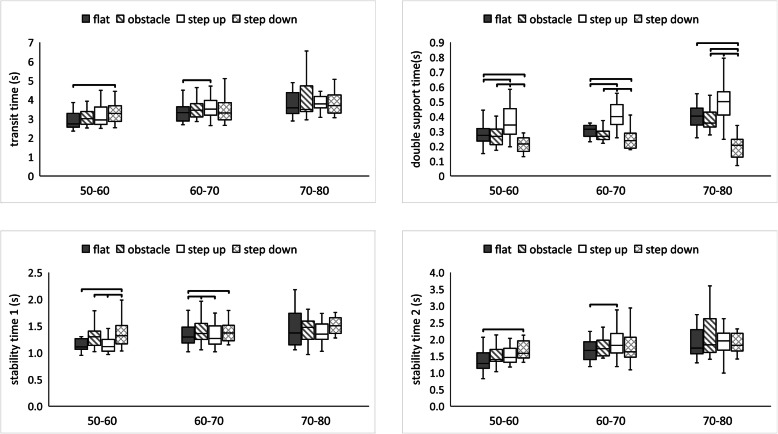
Median value of velocity of COP and standard deviation of COP (minimum, maximum marked as error bars) in the four conditions (1^nd^ and 3^rd^ phase) among groups A, B, and C. The horizontal bars indicate statistically significant differences within the groups (Friedman ANOVA repeated measures with the post hoc test). Abbreviations: flat – unperturbed crossing; obstacle – perturbed crossing

There was a significant impact of testing conditions on v-COP (Chi2 = 24.06; *p* < 0.001) and std-COP (Chi2 = 16.26; p = 0.001) in group A. The subjects obtained the highest values of v-COP during step-up condition and the highest values of std-COP during step-down condition (Fig. 4). There was no significant effect of testing conditions on v-COP and std-COP in group B and C (*p* > 0.05).

## Discussion

Identifying the age-related gait changes will provide insight into the role of aging on postural control during locomotion. In this study, we aimed to determine whether age-associated changes affect postural stability during a transitional task in various conditions. In our case, we provide a procedure which allows the assessment of postural stability among adults of various ages.

### Postural stability during quiet standing before/after transitional task

The findings in our study comprised no significant difference between all three groups of individuals in vCOP and stdCOP during quiet standing before the transitional task. In spite of previous study, where authors noticed differences between young and older adults in COP during quiet standing [15], our results show that these trials were not enough to detect age-related changes in postural control. However, we have noticed differences between people in middle-age and adults over 60 y/o during quiet standing after completing the transitional task. These results confirmed that dynamic task challenging postural control and change the postural stability. Hence, in our study the older adults presented less stable posture after the movement relative to middle-age women, what indicate that adults over 60 y/o exhibit balance deficits. In addition, our analysis showed a significantly greater velocity and standard deviation of COP displacement in quiet standing after the step-up and step-down trials in middle-age women compared to both older adults' groups. As expected, unperturbed transit and crossing obstacle were the simplest tasks for adults in middle-age. This observation suggests that simple motor task do not hamper postural control in younger women. However, the transitional task was very demanding for both older adults' groups independent of trial condition. Hence, it seems that postural deficit are associated with less efficient postural control during more difficult motor tasks.

### Age-related changes during transitional task – preparatory phase

Step initiation consists of an anticipatory postural adjustment (APA) and a stepping phase, both of which are impaired in the older adults. Our results demonstrate that the postural preparation time (S1) was longer in adults over 60 y/o and increased by 17% compared to middle-age subjects. These results suggest that APA might be impaired by age-related physiological changes, which are reflected in reduced somatosensory and visual information. In addition, there is evidence that older people present less variability in muscle activity than younger adults during the anticipatory phase [18], which also confirms that the older adults are unable to respond effectively to balance perturbations [19]. Thus, impaired postural preparation comes with a potential for balance loss in the older population.

### Age-related changes during transitional task – transit phase

In this study, all parameters (TT, S1, S2, DST) increased with age. Also, in previous studies [17] all these parameters were higher in Parkinson’s Disease participants, who present well documented severe balance and gait deficits [20, 21]. In addition, the longer S1, the longer the time of TUG performance. Moreover, the longer the S1, the less points patients gained in BBS, Tinetti test or FRT, which is associated with increased risk of falling and impaired stability. Similar relationships can be noted in TT, which also shows numerous negative correlations with the FRT, Tinetti and BBS [16]. Therefore, we assumed that higher values of the measured parameters indicate impaired postural control during the performance of a transitional task. The transit time was longer by 30% in subjects over 70 y/o, which lead to prolonged DST, S1, and S2. In previous studies, the authors analyzed various gait parameters in older adults, mostly gait speed, step length, and stride length, and rarely double-support time [22, 23]. Some authors [24] observed that adults aged 65–79 y/o present a 20% slower gait speed relative to young adults (20–25 y/o). The decrease in gait speed may reflect a protective adaptation to a perceived threat to stability, as the center of mass must be accelerated from a stationary state, and the relatively small base of support in the first step [24]. Although gait speed has been identified as an important predictor of the onset of immobility and balance disorders among older population [25–27] our results support the idea that double-support time might also be considered as a valuable marker of age-related changes in locomotion. Moreover, there is convincing evidence that DST is highly correlated with gait speed [28]. In our study, DST was longer by 11% among adults 60-70 y/o, but in people over 70 y/o, it was longer by almost 50% compared to middle-age subjects. Since decreased gait speed predicts balance deficits in the older adults, we assumed that a longer DST, which is associated with slower gait speed, indicates impaired postural control during gait among the older women.

### Age-related changes during transitional task – stability regained phase

Additionally, we have noticed a longer regained stability time (S2) in adults over 60 y/o with respect to middle-age participants. The S2 time increased by 18% among adults 60-70 y/o, but in participants over 70 y/o, it increased by 32%. Gait termination changes the gait patterns and thereby threatens the stability of the older adults [29], and the older people also generated less braking force than the middle-aged group [30]; therefore, it takes more time to regain a stable posture after movement. In addition, there is evidence that older women exhibit motor decline, which is due by the lower muscle strength relative to younger women. Hence, the older group exhibited longer balance recovery times compared to the younger women [14, 31]. Our results support these findings; we have noticed that adults over 70 y/o present difficulties with regaining stability after a motor task.

### Age-related changes across the different conditions

In addition, all of the measurements changed across the different conditions. Our results demonstrate that independent of age, TT, S1, and S2 increased while crossing an obstacle. An explanation of these findings consists of the fact that stepping over obstacles increases gait challenges at every age, even in the middle-age population [32, 33]; however, this motor task is still more demanding for the older adults compared to young adults [34]. Previous studies reported that the older people used a more conservative strategy for crossing obstacles relative to young adults, including a slower crossing speed and higher foot clearance while crossing over obstacles [11, 35]. Our study supports these findings, as adults over 60 y/o present a longer TT; in other words, they needed more time for negotiating an obstacle. In addition, TT increased in middle-age adults, which corresponds with recent studies [36]. However, the changes in obstacle crossing in the middle-aged group were observed only in the most challenging tasks (obstacle height 26cm) [26].

Additionally, we noticed that TT increased during step-up and step-down conditions. There is evidence that ascending and descending stairs is a hazardous activity of daily life for adults over 60 y/o [12]. However, our findings show that the postural control alteration while negotiating stairs already occurs in middle-age. The preparatory time and the regained stability time increased in adults over 50 y/o, which is a very important sign, because it may mark the onset of age-related gait changes. Moreover, we observed several changes in DST. Independent of age, we noticed longer DST in the step-up condition compared to flat crossing, which may be an indication of balance disorders imposed on the older women during stair climbing. Prolonged DST provides evidence that older adults need to spend more time on double limb support before transitioning to a single support phase while stepping up. These changes are also already apparent in adults 50-60 y/o, further evidence proving the early signs of age-related gait changes.

In addition, during step initiation before descending stairs, the older adults present decreased stability relative to younger participants [37]. Bosse et al. [37] claimed that the older people generated less braking forces while descending stairs, thus they sway forward like a pendulum instead of controlling the movement of center of mass; this offers evidence that the older adults may not be able to effectively reduce their body sway before the initiation of stepping down. In our case, the most surprising issue was the decreased DST in every age group of adults during the step-down condition. On the basis of the above literature, we assumed that adults with impaired postural control are not able to control the forward COP movement, therefore they shorten the double support phase during the descending step task. Moreover, in the women over 70 y/o every task was difficult; we have not noticed differences between all conditions. Therefore, we assumed that the adults in the 70-80 y/o category would present advanced postural control impairments during transitional task.

### Limitations of the current work and future considerations

A limitation of the study was that our procedure included one step, while in other studies the subjects usually performed a few steps. This one step did not reflect exactly the same conditions as during normal daily life situations. However, our procedure is the simplest and less complicated compared to standard measures of gait initiation. Furthermore, in our study, we investigated only three ranges of age; therefore, we recommend that future studies should include more age groups to better assess the onset of age-related gait changes.

## Conclusions

In conclusion, the proposed procedure is adequate for assessing age-related changes in postural control while completing a transitional task. The analysis of DST enabled the detection of early signs of balance changes in middle-age adults. Furthermore, the older women demonstrated postural impairments before movement initiation and also after a motor task. Additionally, independent of age, the transitional task parameters changed with the increasing difficulty of tasks. In every condition we observed postural changes in the double support time, especially among adults over 60 y/o. However, the most demanding task for all groups of adults was the step-down condition.

## Data Availability

The datasets during and/or analysed during the current study available from the corresponding author on reasonable request.

## Abbreviations

AP: The antero-posterior direction
APA: Anticipatory postural adjustment
COP: Center of foot pressure
DST: Double-support time
S1: Stability time 1
S2: Stability time 2
std-COP: Standard deviation of COP
TT: Transit time
v-COP: COP velocity
